# Effects of virtual reality guided meditation in older adults: the protocol of a pilot randomized controlled trial

**DOI:** 10.3389/fpsyg.2023.1083219

**Published:** 2023-07-28

**Authors:** Karin Cinalioglu, Paola Lavín, Magnus Bein, Myriam Lesage, Johanna Gruber, Jade Se, Syeda Bukhari, Neeti Sasi, Helen Noble, Marie Andree-Bruneau, Cyrille Launay, Justin Sanders, Serge Gauthier, Pedro Rosa, Michael Lifshitz, Bruno J. Battistini, Olivier Beauchet, Bassam Khoury, Stephane Bouchard, Pascal Fallavollita, Ipsit Vahia, Soham Rej, Harmehr Sekhon

**Affiliations:** ^1^Department of Psychiatry, Faculty of Medicine and Health Sciences, McGill University, Montreal, QC, Canada; ^2^Jewish General Hospital, Lady Davis Institute, Montreal, QC, Canada; ^3^GeriPARTy Research Lab, Montreal, QC, Canada; ^4^Faculty of Medicine, Health and Life Sciences, School of Nursing and Midwifery, Queen's University Belfast, Belfast, United Kingdom; ^5^Department of Psychiatry and Addictology, University of Montreal, Montreal, QC, Canada; ^6^Geriatric Institute Research Center, Montreal, QC, Canada; ^7^Department of Medicine, Division of Geriatric Medicine, Jewish General Hospital and Lady Davis Institute for Medical Research, McGill University, Montreal, QC, Canada; ^8^Department of Family Medicine, Faculty of Medicine and Health Sciences, McGill University, Montreal, QC, Canada; ^9^McGill Center for Studies in Aging, Douglas Mental Health University Institute, McGill University, Montreal, QC, Canada; ^10^Faculty of Health Sciences, Interdisciplinary School of Health Sciences, University of Ottawa, Ottawa, ON, Canada; ^11^Department of Educational and Counselling Psychology, Faculty of Education, McGill University, Montreal, QC, Canada; ^12^Department of Psychoeducation and Psychology, Université du Québec Outaouais, Gatineau, QC, Canada; ^13^Division of Geriatric Psychiatry, McLean Hospital, Harvard Medical School, Boston, MA, United States

**Keywords:** virtual reality, mindfulness, meditation, older adults, depression, anxiety

## Abstract

**Background:**

Virtual reality (VR) based meditation has been shown to help increase relaxation and decrease anxiety and depression in younger adults. However, this has not been studied in Randomized Controlled Trials (RCT) in the older adult population. The aim of this RCT is to assess the feasibility and acceptability of a VR-guided meditation intervention for community-dwelling older adults and its effect on stress and mental health.

**Methods:**

We will recruit 30 participants aged ≥ 60 years, whose perceived stress score (PSS) is > 14 (moderate stress), and randomize them 1:1 to the intervention or control waitlist group. The intervention will involve exposure to eight 15-min VR-guided meditation sessions distributed twice weekly for 4-weeks. Two modalities will be offered: in-home and at the hospital.

**Data analysis:**

Baseline and post-intervention assessments will evaluate perceived stress, anxiety, depression, sleep quality, quality of life, and mindfulness skills. Analyses will employ mixed methods repeated ANOVA tests. Qualitative analyses through semi-structured interviews and participant observation will be used to assess participants’ experiences. Study outcomes include: (A) feasibility and acceptability compared to a waitlist control (B) stress, using the Perceived Stress Scale (PSS); (C) anxiety, and depression, using the Generalized Anxiety Disorder-7 (GAD-7) and Patient Health Questionnaire-9 (PHQ-9); (D) insomnia, quality of life and mindfulness skills, using the Athens Insomnia Scale (AIS), Quality of Life Questionnaire (EQ-5D-5L) and Five Facets Mindfulness Questionnaire Short Forms (FFMQ-SF), respectively. We will also measure immersive tendencies, sickness and sense of presence using the Simulator Sickness Questionnaire (SSQ) and the Presence Questionnaire (PQ).

**Discussion:**

Virtual reality-guided meditation could be an acceptable, feasible, safe, and cost-effective novel alternative health intervention for improving older adults’ mental health.

**Clinical trial registration**: *NCT05315609* at https://clinicaltrials.gov.

## 1. Introduction

Stress, anxiety, and depression affect 1 in 4 older adults and cost $838 M to the Canadian healthcare system combined. Stress is associated with increased morbidity and numerous health risks such as increased cardiovascular and metabolic risks, insomnia reduced quality of life in older adults. Current approaches to managing stress and other mental health outcomes in older adults include psychotherapy which can be costly and inaccessible (Moroz et al., 2020), and pharmacotherapy which can induce negative side effects, especially for older adults who are at a greater risk for adverse drug reactions (Tham et al., 2016).

Mindfulness-based interventions have emerged as an alternative treatment option for older adults, with positive health effects reported such as reduced pain symptoms in chronic pain patients and reduced depressive symptoms with medium-to-large effect sizes (Creswell, 2017; Torres-Platas et al., 2019; Wielgosz et al., 2019). Mindfulness meditation may however be difficult for beginner practitioners due to challenges in maintaining attention (Lomas et al., 2015). Virtual reality, an emerging technology that is being widely used in different healthcare settings (e.g., treatment of phobias, post-traumatic stress disorder, pain management, and physical rehabilitation), may address this challenge *via* increased immersion to improve focus during meditation (Seabrook et al., 2020). As older adults are quickly becoming more well-versed with technology (Oh et al., 2021), VR interventions could easily be delivered virtually in community, long-term care homes, or inpatient settings and address barriers such as physical mobility, accessibility, and engagement. Overall, VR has been found to be feasible and acceptable among older adults; a recent meta-analysis of 18 randomized controlled trials (RCTs) found moderate effects of VR video games on older adults’ overall cognitive function and memory, and a large effect on older adults’ depressive outcomes (Yen and Chiu, 2021). These RCTs had sample sizes ranging from *n* = 10 to *n* = 282, and were performed in cognitively healthy older adults living in the community, as well as in older adults with mild cognitive impairments or neurological impairments, such as chronic strokes or Parkinson’s disease, in inpatient or long-term care settings. There is however a gap in the literature, as VR mindfulness interventions have not been studied in older adults (Navarro-Haro et al., 2017; Seifert et al., 2019); one preliminary VR-meditation study in veterans with chronic stress and pain (*n* = 31, average age = 55.2 years) found statistically significant reductions in self-reported pain and stress post-intervention (Liu et al., 2021).While these preliminary findings are promising, a RCT investigating the effects of VR-based mindfulness meditation in older adults has not yet been conducted. This study will assess the feasibility and acceptability of a VR-guided meditation intervention vs. a waitlist control group, and evaluate its effects on stress, depression, and other outcomes versus a waitlist control group.

## 2. Methods

### 2.1. Study design

This is a two-arm, assessor-blinded pilot Randomized Controlled Trial (RCT) of a 4-week virtual-reality (VR) guided meditation intervention vs. waitlist control, evaluating its effectiveness, feasibility, and acceptability for in older adults (*n* = 30, ≥ 60 years of age) who experience stress. This pilot program was created by the GeriPARTy research team at McGill University/ Lady Davis, and ethics approval was received from The Douglas Mental Health Research Institute Research Ethics Board (REB) on March 4th, 2022 (IUSMD-21-48).

### 2.2. Virtual reality meditation group

The intervention group will receive a total of eight 15-min sessions delivered twice weekly over 4 weeks. The videos will be delivered *via* the Oculus Quest 2 VR Headset and will contain nature and mindfulness audio created by the GeriPARTy research group (e.g., social worker, postdoc, and research associates, with > 15 years of experience in meditation practice combined). An interventionist/facilitator will be present in person or by Zoom during the sessions to assist the participant with any technical difficulties or questions. Each session will involve stationary 360° imagery and be conducted in a seated position, be limited to 15 min to help minimize the risk of potential adverse events such as motion sickness and eye strain. Moreover, participants will be able to pause or stop a session any time. At the end of each session participants will answer a brief questionnaire asking about their overall experience of the respective session, and will have an opportunity to ask any questions they may have. Additionally, a summary and reference list will be shared with participants after each session to assist with at-home practice until their next visit.

#### 2.2.1. Virtual reality mindful meditation course

The sessions focus on breath work and body scans, with more guidance/instruction in the earlier sessions which will decrease gradually. The Each session will involve stationary 360° imagery and be conducted in a seated position, be limited to 15 min to help minimize the risk of potential adverse events such as motion sickness and eye strain. Moreover, participants will be reminded of their right to pause or stop the intervention at any time. Participants will also answer a brief questionnaire regarding their overall experience, receive summaries and references of each session for the recommended at-home practice, and will have time to ask questions at the end of each session.

The VR content was developed in eight 15-min sessions in English and French, with scenic stationary 360° nature imagery to simulate a calm natural environment. Additionally, nature sounds were used in the audio recordings along with instructions to guide the meditation to enhance the feelings of relaxation and realness. This meditation intervention focuses on breath meditation and non-judgmental observation. As such, the pre-recorded instructions cover some relevant themes including (1) comfort with silence, (2) learning to notice when the mind is wandering and practicing returning to the meditation object without any judgment, (3) hindrances to meditation, (4) self-compassion, (5) facing pain, (6) meta-meditation, and (7) self-reflection. The multimedia content was developed by the GeriPARTy research team using the GoPro MAX and an external microphone. To make the in-home delivery more feasible, all videos were downloaded onto the Oculus Quest 2 VR headset, thus ensuring that participants did not require high-speed internet also available on YouTube 360°.^1^

### 2.3. Waitlist control

The waitlist-control arm will complete all assessments at baseline and 4-weeks. Participants in this group will receive the meditation program once the RCT is complete.

### 2.4. Participants

Potential participants will be contacted and screened for eligibility. The eligibility criteria for participation in the study are outlined in Table 1. A total of 30 older adults (aged ≥ 60) will be recruited from the community, community organizations, and affiliated hospitals/clinics. Written or recorded verbal informed consent will be obtained from all eligible participants prior to study initiation. Participants will be randomized 1:1 into the treatment group (receiving the VR meditation immediately) or the waitlist control group (receiving the VR meditation after 4 weeks—*primary study endpoint*). Baseline assessments will be completed using REDCap and an introductory digital literacy session will be provided to help the participant familiarize themselves with the technology, and assist with the setup of headsets.

**Table 1:** Eligibility criteria.

Inclusion criteria	Exclusion criteria
(1) Aged ≥ 60 years old	(1) Diagnosis of epilepsy, schizophrenia, brain tumor
(2) Ability to speak in English or French	(2) Recurrent migraines, seizures or traumatic brain injury (TBI) in the last year
(3) Ability to provide consent (4) Residing in the Greater Montreal area	(3) Diagnosis of a substance use disorder in the last year
(5) Having a Perceived Stress Score (PSS) greater than 14	(4) Psychiatric hospitalizations in the last year
	(5) Acute psychotic symptoms or suicidal ideation/intent
	(6) Glaucoma or recovery phase of any eye surgery
	(7) Post-traumatic stress disorder
	(8) Changes to psychoactive medications in the 4 weeks prior to the study
	(9) Severe hearing impairment
	(10) Consumption of alcohol, caffeine, or cannabis within the last 24 h or nicotine within the last 15 min before a session

### 2.5. Study settings

This intervention will be offered in a remote or in-person modality in accordance with the government regulations for COVID-19 at the time of the study. Participants will be allocated at a 1:1 ratio to the VR meditation intervention or the waitlist control group by an external researcher using computer randomization. Participants will be reminded to not disclose their group allocation prior to study enrollment as all research assessors will be blinded to the randomization of participants.

#### 2.5.1. Remote/in-home delivery

Prior to study initiation, participants in the remote/in-home delivery arm will receive an Oculus Quest 2 VR device treated with UV-sanitizing Cleanbox technology and a package with eight brief handouts to supplement each session of the VR intervention with information on mindfulness meditation as well as a journal to record the number of minutes practiced outside the VR intervention session. Furthermore, an introductory digital literacy session will be offered through Zoom to help participants familiarize themselves with the technology a week before the first session.

#### 2.5.2. In-person/hospital delivery

The in-person delivery of the program will take place at the Douglas Hospital and Lady Davis Institute for Medical Research/ Jewish General Hospital, Montreal, QC, Canada. All COVID protocols will be followed (e.g., masks, hand sanitizer), and the devices will be sanitized using the Cleanbox technology, UV sanitizing device. Additionally, high-contact surfaces such as chair arms, and doorknobs, will be sanitized between participants. Participants will receive a package with eight brief handouts to supplement each VR intervention with information on mindfulness meditation and a journal to record the number of minutes practiced at home at the end of the first session.

### 2.6. Study outcomes

All quantitative outcomes (primary, secondary, exploratory) will be assessed at baseline and 4 weeks *(primary study endpoint)*, in-person or remotely by phone. All assessments will be administered by an assessor who is blinded to group allocation.

#### 2.6.1. Primary outcome

The Perceived Stress Scale (PSS; Reis et al., 2010), is a 14-item scale used to measure the degree to which life events are experienced and appraised as stressful. Respondents are to indicate how often they have felt certain ways in the past month, with responses ranging from 0 (never) to 4 (very often).

#### 2.6.2. Secondary outcomes

The secondary outcomes anxiety and depression will be measured using the Generalized Anxiety Disorder scale (GAD-7) and the Patient Health Questionnaire (PHQ-9), respectively. The GAD-7 (Williams, 2014) is a 7-item scale that measures symptoms of anxiety present in the previous 2 weeks. Respondents’ scores can range from 0 (not at all sure) to 3 (nearly every day). Items include “Not being able to stop or control worrying” and “Being so restless that it’s hard to sit still.” The PHQ-9 (Kroenke et al., 2001) is a 9-item self-report questionnaire used to diagnose depression and assess symptom severity.

#### 2.6.3. Additional measures and covariates

Exploratory outcome measures will include (1) EuroQol-5 Dimension (EQ-5D-5L; Herdman et al., 2011), a standardized measure of health-related quality of life. It is a 5-component scale including mobility, self-care, usual activities, pain/discomfort, and anxiety/depression; (2) Athens Insomnia Scale (AIS; Soldatos et al., 2000) is an 8-item, self-report scale to assess sleep quality, assessing sleep induction, awakenings during the night, final awakening earlier than desired, total sleep duration, and sense of well-being during the day, sleepiness, and daytime functioning; (3) the Five Facet Mindfulness Questionnaire—Short Form (FFMQ-SF; Bohlmeijer et al., 2011) is a 24-item scale that is used to assess whether mindfulness is related to a decrease in clinical symptoms of depression, anxiety, and stress; (4) the UCLA-3 Item Loneliness Scale (Russell, 1996) measures the subjective experience of loneliness on a four-point Likert scale; (5) the Simulator Sickness Questionnaire (SSQ) Simulator Sickness Questionnaire (Kennedy et al., 1993) is a 16-item scale that is widely used to assess simulator sickness when using virtual reality. (6) the Witmer and Singer Presence Questionnaire (Witmer and Singer, 1998) is a self-report presence measure to evaluate experiential aspects of immersive technology. The latter two questionnaires will be administered at each session in the intervention group. Furthermore, participants will answer a brief questionnaire regarding their overall experience, receive summaries and references of each session for the recommended at-home practice, and will have time to ask questions at the end of each session. Additionally, qualitative data about participants’ experiences (e.g., perceptions, challenges, perceived usability), will be collected through semi-structured interviews and participant observation.

### 2.7. Data collection and analysis

The REDCap platform will be used for all data collection and storage. Descriptive analyses will be conducted for the demographics data and group differences at baseline will be analyzed using a Chi-square. 1) A mixed methods repeated measures ANOVA [(within-subject (time) × between-subject (group)] factors will be conducted to evaluate changes in stress (PSS), anxiety (GAD-7), and depression (PHQ-9) scores from baseline to 4-weeks (*primary study endpoint*) in the intervention group vs. waitlist control.

Additional analysis of covariance (ANCOVAs) will control for variables that differ between groups at baseline. All ANOVAs will be tested at two-tailed alpha = 0.05. An additional sensitivity analysis two-way ANOVA will be performed to compare participants who (1) engaged in daily home practice (average of ≥ 10 min, ≥ 4 days/week). Missing data will be handled using the last observation carried forward (LOCF). Analyses will be performed using R statistical software. Additional subgroup analyses will also compare the primary outcome (PSS) between important subgroups [e.g., (remote vs. in-person) and baseline stress (higher vs. lower)].

#### 2.7.1. Qualitative evaluation and analysis

Qualitative evaluation of the intervention will be conducted by an expert qualitative researcher and will involve participant observation and semi-structured interviews to gain further insight into the feasibility and acceptability of the intervention. The interviews will be held at 4 weeks (*primary study endpoint*), and use an interview guide with open-ended questions which will take between 30 and 50 min to complete depending on the participant. Participant observation will also be conducted with 8–10 clients/service users during the intervention sessions. Additionally, the interventionists will take observational notes on participants’ engagement and interactions with the technology throughout the intervention which will enhance the interpretation of qualitative findings. The qualitative part of the study will analyze service users’ perceptions, experiences, engagement, and challenges throughout the study. Participant observation, conducted throughout the intervention will provide a context for the interviews which will be conducted after the end of the intervention. During the participant observation sessions, we will focus on observing and understanding the user’s perception, engagement, usage, expressions, physical movements, feedback, and barriers while using VR technology for guided meditation. The focus of data collection in interviews will remain on the perceptions and experiences of the users/participants and a semi-structured, open-ended interview guide will help lead the discussions. We will use an inductive coding approach for sorting and coding the data until themes start to emerge. The process will link themes to data, keep them empirically grounded, and will also allow research findings to emerge from the most frequent, dominant, or important themes (Miles and Huberman, 1994). Triangulation of data (interviews and observational notes) will be implemented to cross-check and verify themes and overall findings. For coding and data analysis, NVIVO12 (Dhakal, 2022) software will be used.

## 3. Discussion

While mindfulness meditation interventions in older adults have been shown to improve physical and mental health outcomes, they may be difficult for beginner practitioners due to challenges in maintaining attention. VR can address this challenge *via* increasing immersion and decreasing distractions to improve focus during meditation, and can further address barriers to care often faced by older adults, such as reduced physical mobility, accessibility, engagement, and motivation.

The proposed study is the first RCT exploring the effects of VR-based mindfulness meditation in older adults and presents a safe potential intervention to address stress and depression in community-dwelling older adults. If successful, this intervention may benefit the society and the healthcare system by potentially decreasing the number of Canadians suffering from chronic stress, and ultimately decrease the economic burden on the healthcare system as it could be delivered in an at-home setting and become an easily scalable and cost-effective novel alternative intervention to promote a better mental health and well-being in older adults. If the proposed study is found feasible, future directions may include assessing the feasibility of similar interventions in online or in-person group settings. The interactive nature of VR would also allow for helping address the social needs of older adults who have limited mobility or live in rural regions. The results of this study, regardless of the outcome, will provide essential pilot data for future larger studies investigating similar interventions in older adult populations. It is also possible, however, that participants will experience partial or no improvement.

## Ethics statement

Ethics approval for this study was received from The Douglas Mental Health Research Institute ethics review board (ERB) on March 4th, 2022. The patients/participants provided their written informed consent to participate in this study.

## Author contributions

KC, PL, HS, and SR: conceptualization and methodology. KC, PL, MB, and HS: draft preparation and writing and editing. MyL, JG, JaS, StB, NS, HN, MA-B, CL, JuS, SG, PR, MiL, BB, OB, BK, SyB, PF, and IV have substantially contributed to the preparation, critical review, commentary revision, and approval of the manuscript.

## Funding

This research was funded by The Fonds de Recherche du Québec (FRQS) Grant #2022-VIAP-308195, Canadian Institutes of Health Research [CIHR] grants #PJT-175191, #169696, and Charitable donations from Doggone Foundation, and JGH foundation. HS was supported by the CIHR fellowship and AGE-WELL Award. SR received a salary award from FRQS.

## Conflict of interest

SR owns shares in Alfred-Health, is on a steering committee for Abbvie, and has received an operating grant from Mitacs. StB is the President of, and owns equity in, Cliniques et Développement In Virtuo, a spin-off from his university that uses virtual reality and distributes virtual environments.

The remaining authors declare that the research was conducted in the absence of any commercial or financial relationships that could be construed as a potential conflict of interest.

## Publisher’s note

All claims expressed in this article are solely those of the authors and do not necessarily represent those of their affiliated organizations, or those of the publisher, the editors and the reviewers. Any product that may be evaluated in this article, or claim that may be made by its manufacturer, is not guaranteed or endorsed by the publisher.

## Footnotes

^1^ https://www.youtube.com/channel/UC7PEflPp1n2QuaxGTYzCHvg/videos
